# Gender disparities in Swedish specialized nephrology care

**DOI:** 10.1093/ckj/sfag302

**Published:** 2026-09-01

**Authors:** Frida Welander, Barbara Salzinger, Stefan H Jacobson, Juan-Jesus Carerro, Marie Evans

**Affiliations:** Division of Renal Medicine, Department of Clinical Science Intervention and Technology at Karolinska Institutet, Stockholm, Sweden; Department of Public Health and Clinical Medicine, Department of Research and Development-Sundsvall, Umeå University, Sundsvall, Sweden; Division of Nephrology, Department of Clinical Sciences, Karolinska Institutet Danderyd Hospital, Stockholm, Sweden; Division of Nephrology, Department of Clinical Sciences, Karolinska Institutet Danderyd Hospital, Stockholm, Sweden; Division of Nephrology, Department of Clinical Sciences, Karolinska Institutet Danderyd Hospital, Stockholm, Sweden; Department of Medical Epidemiology and Biostatistics, Karolinska Institutet, Stockholm, Sweden; Division of Renal Medicine, Department of Clinical Science Intervention and Technology at Karolinska Institutet, Stockholm, Sweden

**Keywords:** chronic kidney disease, gender disparities, kidney replacement therapy, quality of care

## Abstract

**Background:**

Although women have more prevalent chronic kidney disease (CKD) they less often have recommended therapy or a documented CKD diagnosis in primary care. This study investigates whether gender disparities remain in specialized nephrology care as a possible expression of gender bias.

**Methods:**

Patients with incident estimated glomerular filtration rate (eGFR) <30 mL/min/1.73 m^2^ registered in the Swedish Renal Registry between 2010 and 2020 were included. Logistic regression investigated gender differences in having a CKD diagnosis, kidney biopsy, blood pressure (BP) <140/90 mmHg and guideline-recommended medications. Gender disparities regarding kidney replacement therapy (KRT) initiation, including preemptive kidney transplantation and initiating dialysis with a planned access, were investigated using flexible parametric regression and Cox regression.

**Results:**

Of 25 478 included patients 37% were female. Women were less likely to have a CKD diagnosis [odds ratio (OR) 0.81, 95% confidence interval 0.73–0.90], a renin–angiotensin–aldosterone inhibitor (OR 0.94, 0.89–0.99), lipid-lowering agent (OR 0.93, 0.88–0.99) or BP <140/90 mmHg (OR 0.91, 0.86–0.97). Women had borderline-significantly lower odds of having a kidney biopsy (OR 0.93, 0.86–1.00). Women were less likely to initiate KRT, [hazard ratio (HR) 0.75, 0.70–0.80] or to initiate dialysis with a planned access (HR 0.82, 0.78–0.85). No significant gender differences were seen regarding preemptive transplantation and conservative kidney management decision. Women less often died before KRT initiation (HR 0.81, 0.77–0.85).

**Conclusions:**

Swedish women with advanced CKD in specialized nephrology remain underdiagnosed, undertreated and less likely to reach BP targets. Although these gender differences did not translate to increased KRT and mortality rates, they highlight the need for nephrologists to maintain awareness and strive for health equity.

## Graphical Abstract

**Graphical Abstract:**
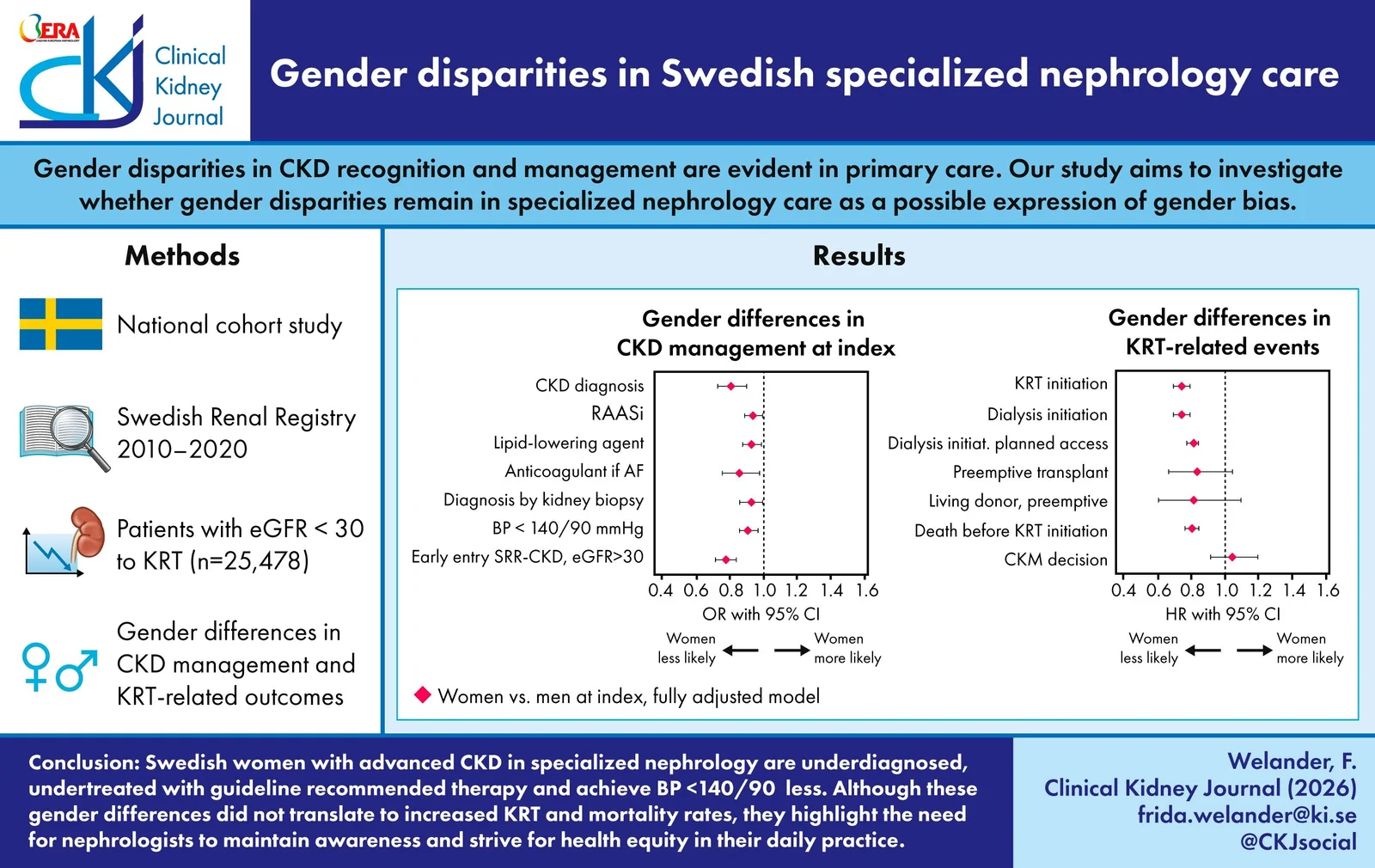

KEY LEARNING POINTS **What was known:**

- Women with chronic kidney disease (CKD) in primary care have lower chances of receiving guideline-recommended care.

**This study adds:**

- Gender differences persist in the management of patients with advanced CKD in specialized nephrology care as registered in the nationwide Swedish Renal Registry.
- Women with incident estimated glomerular filtration rate <30 mL/min/1.73 m^2^ in nephrology care have lower chances of having a documented CKD diagnosis, guideline-recommended therapy or blood pressure <140/90 mmHg.
- Despite receiving less guideline-recommended care, women have lower KRT and mortality rates.

**Potential impact:**

- There are gender differences Swedish nephrology care.
- Nephrologists need to maintain awareness and strive for health equity in their daily work.

## INTRODUCTION

Health equity is a core goal of the World Health Organization, aligning with United Nations’ Sustainable Development Goals for 2030. It is achieved when individuals can attain their full health potential irrespective of economics, demographics, social group, sex, gender, ethnicity, disability or sexual orientation [1].

Chronic kidney disease (CKD) causes substantial global mortality and morbidity, and is predicted to be the fifth leading cause of death by 2040 [2]. CKD is more prevalent in women and early recognition offers the possibility to slow progression, delay kidney failure and prevent cardiovascular disease [3, 4]. The cornerstones of CKD management include testing for albuminuria with urinary albumin/creatinine ratio (ACR), blood pressure (BP) control, and guideline-recommended therapy including renin–angiotensin–aldosterone inhibitors (RAASi), statins and sodium-glucose cotransporter 2 inhibitors [5]. Many primary kidney diseases are treatable but require correct diagnosis, often by a kidney biopsy. A Swedish study showed that women with incident estimated glomerular filtration rate (eGFR) <60 mL/min/1.73 m^2^ in primary care were less likely to be recognized with a CKD diagnosis, evaluated by a nephrologist or have recommended therapy such as RAASi, a pattern also shown in other countries [6–9]. It is not known whether these gender disparities remain after referral to nephrology care.

Although CKD is more prevalent in women, men are more likely to progress to kidney failure [3, 10]. Over 60% of the patients initiating kidney replacement therapy (KRT) are men, largely due to faster disease progression and higher levels of albuminuria [11]. Lower rates of female KRT initiation could also reflect fewer women being referred to nephrology or that more women opt for conservative kidney management (CKM) [12]. Gender disparities in access to KRT are well described globally. However, investigating this in Sweden, a country ranked highly for gender equality, is of particular interest [13].

The Swedish Renal Registry Chronic Kidney Disease (SRR-CKD), a comprehensive national registry of nephrology-referred CKD patients, provides an opportunity to examine gender differences throughout CKD progression. We hypothesize that women with advanced CKD in Swedish specialized nephrology care are less likely to receive recommended management and guideline-recommended initiation of KRT including preemptive kidney transplantation.

## MATERIALS AND METHODS

This was a cohort register study adhering to the Declaration of Helsinki approved by the Swedish Ethical Review Authority (2018/1591–31/2). Personal consent was waived due to the register-based design.

### Cohort

The cohort was derived from the SRR-CKD, a nationwide registry prospectively following patients with advanced CKD. The registry systematically includes patients with eGFR <30 mL/min/1.73 m^2^ (some clinics use an earlier eGFR threshold, such as <60 mL/min/1.73 m^2^) from 98% of all Swedish nephrology clinics and reporting variables such as eGFR, BP and laboratory measurements are mandatory [14]. Patients entered between 1 January 2010 and 31 December 2020 with index date defined as the first documented eGFR <30 mL/min/1.73 m^2^ (Chronic Kidney Disease Epidemiology Collaboration [15]). Follow-up continued until KRT initiation, death or 31 December 2020. Exclusion criteria were age <18 years, prior KRT or death/KRT on index date.

### Exposure

The exposure was gender (man/woman). Gender is a social construct and does not need to align with biological sex. If a Swedish person decides to legally transition, their unique personal identity number will change, where the second last digit represents legal gender. We retrieved gender from the personal identity number representing legal, not self-reported, gender.

### Outcomes and covariates

Using the personal identity number, data from SRR-CKD were linked to other health registers. CKD diagnoses and relevant comorbidities were obtained from The Swedish National Patients Register (NPR), a register with an almost complete coverage of International Classification of Diseases, 10th revision (ICD-10) codes of all inpatient and specialized outpatient care. The covariate hypertension was identified from NPR or if the index BP was ≥140/90 mmHg in SRR-CKD. The Swedish Prescribed Drug Register has complete coverage of all drug dispenses by Swedish pharmacies and provided the medication data.

This study was divided into two parts: firstly, a cross-sectional analysis using retrospective data up until and including the index date, and secondly a follow-up part of time to event for several prespecified outcomes. In the first part of the analysis, outcomes potentially affected by gender bias were selected based on contemporary guidelines and what we considered as high-value care [16]. The outcomes selected were: (i) a documented CKD diagnosis (N18, ICD-10) ever in NPR; (ii) a documented kidney biopsy, excluding diagnoses not requiring biopsy (excluded diagnoses in Supplementary data, Table S1) based upon procedure code in NPR or as per definition according to the European Renal Association (ERA) Primary Renal Disease code in SRR-CKD; (iii) RAASi treatment; (iv) lipid-lowering agent treatment (if age >50 years); (v) oral anticoagulant treatment if atrial fibrillation (AF) and CHA_2_DS_2_-VASc ≥2 (defined as a drug dispensation within 6 months) [17] [CHA_2_DS_2_-VASc = congestive heart failure, hypertension, age ≥75 years (doubled), diabetes mellitus, prior stroke or transient ischaemic attack (doubled), vascular disease, age 65–74 years, female]; (vi) documented BP <140/90 mmHg at an out-patient nephrology visit registered in SRR-CKD within 6 months before index; and (vii) early versus late entry into SRR-CKD (defined as having first eGFR ≥30 mL/min/1.73 m^2^ at first registration).

In the second part of the analysis, KRT-related outcomes potentially influenced by gender bias were selected based on guidelines: KRT initiation, dialysis initiation, dialysis initiation with planned access, preemptive kidney transplantation, living donor (LD) preemptive transplantation, death before KRT initiation and a documented CKM decision [18, 19]. All outcome data were collected from the SRR.

### Statistical analysis

All analyses were performed with Stata, version 18. Baseline data was presented with counts (percentage) for categorial and median [interquartile range (IQR)] for continuous variables (ICD-10 specification in Supplementary data, Table S2). For the first part of the study, we used logistic regression models to estimate the odds ratios (OR) with 95% confidence interval (95% CI) for women versus men, unadjusted and adjusted. While sex has no true confounders, there are several mediators of gender differences related to our outcomes and adjustments were predefined based on consensus by the authors and applied per outcome. All adjusted models include sex (legal gender), age and eGFR (cubic splines, 5 knots), pulse pressure and logarithmic (log) ACR (modelled linearly), hypertension, congestive heart failure, diabetes mellitus, ischaemic heart disease, stroke and vascular disease. The outcome anticoagulant treatment was also adjusted for log-haemoglobin (linearly) and history of a previous major bleeding. The outcome kidney biopsy was additionally adjusted for anticoagulant treatment, previous major bleeding and log-haemoglobin.

For the second part of the study, absolute risk of the events was predicted at 3 years with flexible parametric regression and 95% CI calculated with bootstrapping, 200 repetitions. This risk was presented unadjusted and adjusted. Aalen–Johansen curves estimated the cumulative incidence function with *P*-values according to Pepe–Mori [20]. Patients were followed from inclusion until KRT initiation, CKM decision, death or 31 December 2020, whichever occurred first. Hazard ratios (HR) were calculated using cause-specific Cox proportional hazards regression; these analyses were adjusted for the same covariates as listed above with addition of log-phosphate and log-haemoglobin. Time to CKM decision was analysed with the same adjustments and additionally adjusted for history of dementia and cancer the last 5 years. Missing data (missingness table presented in Supplementary data, Table S3) were handled using multiple imputation by chained equations in Stata, generating 20 imputed datasets with linear regression models. All survival analyses are censored for the competing events death and CKM decision, except the analyses where these events are the primary outcomes. For survival analysis of CKM decision, a documented KRT decision or initiation of KRT are treated as competing events.

Three sensitivity analyses were prespecified: complete cases (patients with no missing covariates for the specific analysis) and two index time periods (2010–15 and 2016–20). A variable with large missingness is ACR (43% missingness); this is due to ACR first becoming mandatory to report to SRR-CKD in 2015. Investigating time periods was done due to a theory that gender differences might be more pronounced in the early time period, since awareness of the topic has increased with time. A post-hoc analysis was added investigating time to KRT outcomes exclusively in patients with a documented KRT decision. In a second post-hoc analysis we investigated the robustness of the CKD diagnosis outcome adding ICD-10 codes N00–N19 and Q61.

## RESULTS

### Patients’ characteristics

The register contained 41 133 unique individuals. Of these, 25 645 were aged >18 years and had a registered visit with eGFR <30 mL/min/1.73 m^2^ between 1 January 2010 and 31 December 2020. Among patients eligible for inclusion, 166 were excluded due to previous KRT treatment and 1 was excluded due to death on index, leaving 25 478 included patients with median age 74 years, 37% female (Table 1). At baseline, women and men had similar eGFR (22 vs 23 mL/min/1.73 m^2^). Men more often had diabetes, hypertension and heart disease, while more women had chronic obstructive pulmonary disease/asthma and psychiatric disease.

**Table 1:** Baseline characteristics.

	Total, *n* = 25 478	Women, *n* = 9440	Men, *n* = 16 038	*P*-value
Age (years), median (IQR)	74 (65–81)	74 (65–81)	74 (65–80)	.052
eGFR (mL/min/1.73 m^2^) at index (first visit eGFR <30), median (IQR)	23 (17–27)	22 (17–26)	23 (18–27)	<.001
eGFR categories, *n* (%)				<.001
CKD G4 (eGFR 29–15 mL/min/1.73 m^2^)	21 248 (83)	7712 (82)	13 536 (84)	
CKD G5 (eGFR <15 mL/min/1.73 m^2^)	4230 (17)	1728 (18)	2502 (16)	
U-ACR (mg/mmol), median (IQR)	34 (6–149)	20 (4–120)	45 (9–163)	<.001
U-ACR categories (mg/mmol), *n* (%)				<.001
<30	6886 (27)	2857 (30)	4030 (25)	
30–149	3940 (15)	1168 (12)	2772 (17)	
150–299	1896 (7)	534 (6)	1362 (8)	
≥300	12 756 (50)	4882(52)	7878 (49)	
P-Albumin (g/L), median (IQR)	37 (34–40)	37 (34–40)	37 (34–40)	.84
CRP (mg/L), median (IQR)	5 (2–10)	5 (2–10)	5 (2–10)	.40
Calcium (mmol/L), median (IQR)	2 (2–2)	2 (2–2)	2 (2–2)	<.001
PTH (mmol/L), median (IQR)	14 (9–22)	14 (9–22)	14 (9–22)	.33
Creatinine (µmol/L), median (IQR)	223 (193–275)	194 (166–242)	237 (207–292)	<.001
Systolic BP (mmHg), median (IQR)	140 (125–152)	140 (125–151)	140 (125–152)	.27
Diastolic BP (mmHg), median (IQR)	78 (70–84)	76 (69–82)	79 (70–85)	<.001
Body mass index (kg/m^2^), median (IQR)	27 (24–32)	28 (24–32)	27 (24–31)	<.001
Haemoglobin (g/L), median (IQR)	119 (109–131)	116 (107–126)	121 (110–133)	<.001
Phosphate (mmol/L), median (IQR)	1.3 (1.1–1.4)	1.3 (1.1–1.5)	1.2 (1.1–1.4)	<.001
Comorbidities, *n* (%)				
Hypertension	20 996 (82)	7649 (81)	13 347 (83)	<.001
Diabetes mellitus	10 828 (42)	3857 (41)	6971 (43)	<.001
AF	5134 (20)	1671 (18)	3463 (22)	<.001
Congestive heart failure	6856 (27)	2427 (26)	4429 (28)	<.001
Stroke	3046 (12)	960 (10)	2086 (13)	<.001
Systemic embolism	301 (1)	109 (1)	192 (1)	.76
Venous thromboembolism	1018 (4)	407 (4)	611 (4)	.048
COPD or asthma	3302 (13)	1432 (15)	1870 (12)	<.001
Vascular disease	6514 (26)	2012 (21)	4502 (28)	<.001
Ischaemic heart disease	7348 (29)	2229 (24)	5119 (32)	<.001
Dementia	308 (1)	113 (1)	195 (1)	.89
Psychiatric disease	2399 (9)	1224 (13)	1175 (7)	<.001
Major bleeding	3815 (15)	1214 (13)	2601 (16)	<.001
Cancer	4203 (16)	1190 (13)	3012 (19)	<.001
CHA_2_DS_2_-VASc, median (IQR)	4 (2–5)	4 (3–5)	3 (2–5)	<.001
Dispensed medications 6 months prior to index, *n* (%)				
RAASi	16 647 (65)	5985 (63)	10 662 (66)	<.001
Beta-blockers	16 216 (64)	5929 (63)	10 287 (64)	.033
Calcium inhibitors	14 698 (58)	5129 (54)	9569 (60)	<.001
Diuretics	16 255 (64)	6229 (66)	10 026 (63)	<.001
Number of antihypertensives, median (IQR)	2 (1–3)	2 (1–2)	2 (1–3)	<.001
Lipid-lowering agents	13 792 (54)	4743 (50)	9049 (56)	<.001
Insulin	7346 (29)	2606 (28)	4740 (30)	<.001
Metformin	710 (3)	308 (3)	402 (3)	<.001
GLP1 analogues	403 (2)	129 (1)	274 (2)	.035
SGLT2 inhibitors	69 (0.3)	17 (0.2)	52 (0.3)	.033
Anticoagulants (VKA or DOAC)	4273 (17)	1372 (15)	2901 (18)	<.001
VKA	3317 (13)	1017 (11)	2300 (14)	<.001
DOAC	1059 (4)	388 (4)	671 (4)	.78
Acetylsalicylic acid	8970 (35)	2993 (32)	5977 (37)	.035
Erythropoietin-stimulating agents	6921 (27)	2699 (29)	4222 (26)	<.001

The table shows baseline characteristics at first registered outpatient visit after eGFR drops <30 mL/min/1.73 m^2^. Data are presented as median (IQR) for continuous measures, and *n* (%) for categorical measures.

a Cancer within 5 years, non-melanoma skin cancers excluded.

b CHA_2_DS_2_-VASc [congestive heart failure, hypertension, age ≥75 years (2 points), diabetes mellitus, stroke or venous thromboembolism (2 points), vascular disease, age 65–74, sex (female)]

U-ACR, urine ACR; CRP, C-reactive protein; PTH, parathyroid hormone; COPD chronic obstructive pulmonary disease; AF atrial fibrillation; GLP1, glucagon-like peptide-1; SGLT2, sodium-glucose cotransporter; VKA, vitamin K antagonists; DOAC, direct oral anticoagulants.

### Gender differences in CKD management at index

At first registered nephrology outpatient visit with eGFR <30 mL/min/1.73 m^2^, 93% had a documented CKD diagnosis with lower odds for women compared with men (adjusted OR 0.81, 95% CI 0.73–0.90) (Table 2, Fig. 1A). Women were less likely to receive RAASi (0.94, 0.89–0.99), lipid-lowering agents (0.93, 0.88–0.99) of anticoagulants (0.86, 0.76–0.98) if AF, and had borderline lower odds of being diagnosed by kidney biopsy (0.93, 0.86–1.00). Women displayed lower odds of achieving BP <140/90 mmHg (0.91, 0.86–0.97) and of being entered early in SRR-CKD (eGFR ≥30 mL/min/1.73 m^2^ at the first documented visit) (0.78, 0.72–0.84).

**Figure 1:**
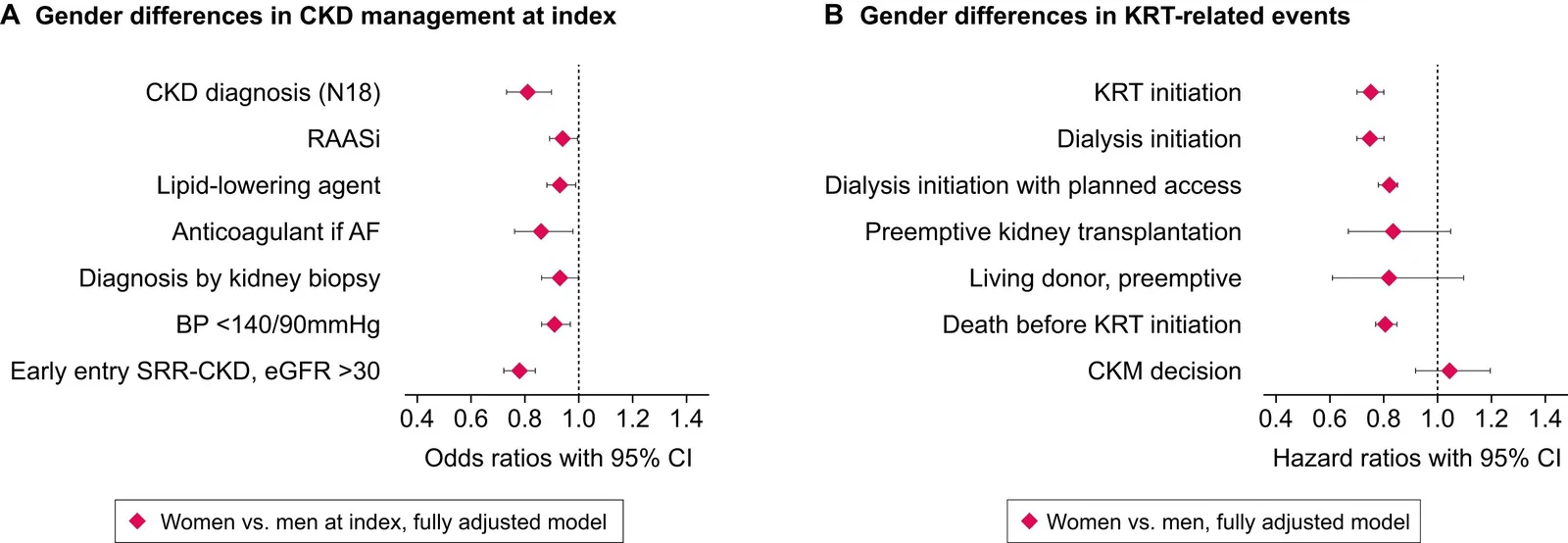
(**A**) Gender differences (women versus men) in incident patients at the first outpatient visit with eGFR <30 mL/min/1.73 m^2^ registered in SRR-CKD. Results presented with OR and 95% CI. Adjusted models include sex (legal gender), age and eGFR (cubic splines, 5 knots), pulse pressure and logarithmic (log) ACR (modelled linearly), hypertension, congestive heart failure, diabetes mellitus, ischaemic heart disease, stroke and vascular disease. The outcome anticoagulant treatment was also adjusted for log-haemoglobin (linearly) and history of previous major bleeding. The outcome kidney biopsy was additionally adjusted for anticoagulant treatment, previous major bleeding and log-haemoglobin. (**B**) Gender differences in KRT-related events (women versus men) with HR and 95% CI from a fully adjusted cause-specific Cox proportional hazards regression analysis. Adjusted for sex, age-splines, eGFR-splines, pulse pressure, ACR (logarithmic), haemoglobin (logarithmic), phosphate (logarithmic), hypertension, congestive heart failure, diabetes mellitus, ischaemic heart disease, stroke and vascular disease. Conservative kidney management decision was also adjusted for a diagnosis of cancer the last 5 years and dementia. Patients were followed from inclusion until KRT initiation, CKM decision, death or 31 December 2020, which ever occurred first. Missing data were handled using multiple imputation by chained equations in Stata, generating 20 imputed datasets with linear regression models.

**Table 2:** Gender differences in CKD management at index.

	Women n, (% of eligible); men n, (% of eligible)	Unadjusted OR (95% CI)	Adjusted OR (95% CI)
Documented CKD diagnosis (N18)	8661 (92); 14 985 (93)	**0.78 (0.71–0.86)**	**0.81 (0.73–0.90)**
RAASi	5985 (63); 10 662 (66)	**0.87 (0.83–0.92)**	**0.94 (0.89–0.99)**
Lipid-lowering agent if aged >50 years	4498 (52); 8542 (58)	**0.78 (0.74–0.82)**	**0.93 (0.88–0.99)**
Anticoagulants if AF and CHA_2_DS_2_-VASc ≥2	1051 (63); 2244 (66)	**0.87 (0.77–0.99)**	**0.86 (0.76–0.98)**
Diagnosis by kidney biopsy	1515 (18); 2823 (20)	**0.89 (0.83–0.95)**	0.93 (0.86–1.00)
BP <140/90 mmHg	4184 (47); 7106 (47)	1.01 (0.96–1.07)	**0.91 (0.86–0.97)**
Early entry in SRR-CKD, first eGFR ≥30 mL/min/1.73 m^2^	1519 (16); 3367 (21)	**0.72 (0.68–0.77)**	**0.78 (0.72–0.84)**

CHA_2_DS_2_-VASc: congestive heart failure, hypertension, age ≥75 years (doubled), diabetes mellitus, prior stroke or transient ischaemic attack (doubled), vascular disease, age 65–74 years, female.

The table shows gender differences (women versus men) in incident patients at the first outpatient visit with eGFR <30 mL/min/1.73 m^2^ registered in SRR-CKD. Results are presented as OR and 95% CI, unadjusted as well as adjusted. All adjusted models include sex (legal gender), age and eGFR (cubic splines, 5 knots), pulse pressure and log-ACR (modelled linearly), hypertension, congestive heart failure, diabetes mellitus, ischaemic heart disease, stroke and vascular disease. The outcome anticoagulant treatment was also adjusted for log-haemoglobin (linearly) and history of previous major bleeding. The outcome kidney biopsy was additionally adjusted for anticoagulant treatment, previous major bleeding and log-haemoglobin. Missing data were handled using multiple imputation by chained equations in Stata, generating 20 imputed datasets with linear regression models.

a Bold values have significant confidence intervals

### Gender differences in KRT-related outcomes

During follow-up (median 2.3 years, IQR 1.0–4.1), 5914 patients initiated KRT: 5538 (94%) via dialysis and 376 (6%) via preemptive transplantation; 33% were women.

### Cumulative incidence function and absolute risks

Gender differences in the KRT-related outcomes are presented graphically as the cumulative incidence function (Fig. 2).

**Figure 2:**
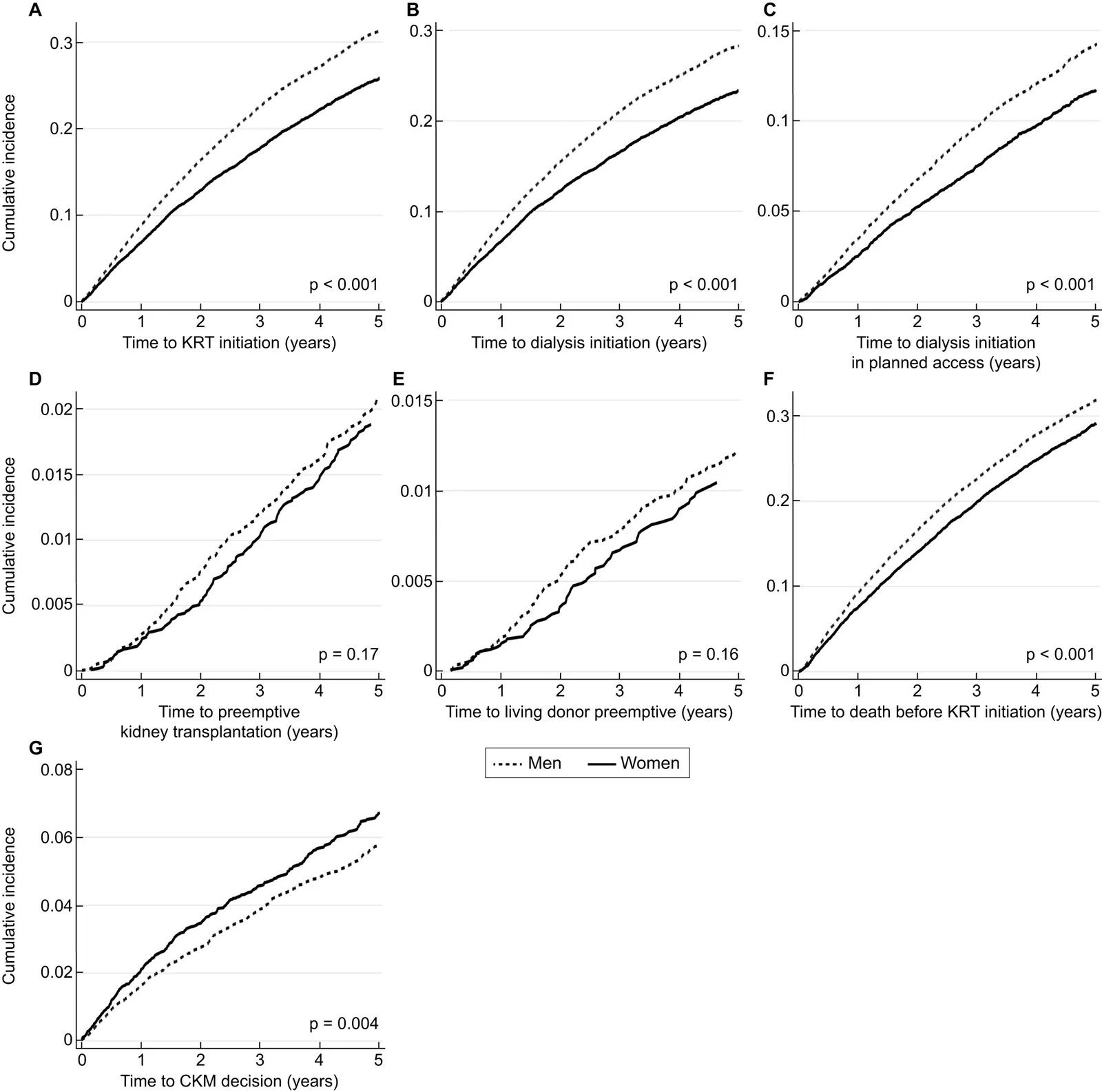
Cumulative incidence function curves for the KRT-related events. The figure shows the unadjusted cumulative incidence function (CIF) curves (Aalen–Johanssen) for outcomes. *P*-values calculated with Pepe–Mori test. (**A**) Time to KRT initiation. (**B**) Time to dialysis initiation. (**C**) Time to dialysis initiation with planned access. (**D**) Time to preemptive transplantation. (**E**) Time to LD preemptive transplantation. (**F**) Time to death before KRT initiation. (**G**) Time to CKM decision. Larger versions of panels (A) to (G) can be found in the Supplementary data, Fig. S3A–G.

The corresponding absolute risks were predicted at 3 years using adjusted flexible parametric regression (Table 3), showing significantly lower risk of KRT initiation for women (*P* < .001) as well as dialysis initiation, including initiation in planned access (*P* < .001). The risk of death was also significantly lower for women (*P* < .001) There were no significant gender differences in the chances of being preemptively transplanted (including LD transplantation). Unadjusted, there was a higher absolute risk for women of having a documented CKM decision (*P* = .02), however this was no longer significant after adjustments (*P* = .16).

**Table 3:** Gender differences in KRT-related events.

Outcome	Women *n*, (% of eligible); men *n*, (% of eligible)	Absolute risk (%) at 3 years, women; men	Risk difference (females − males) (95% CI); *P*-value	Absolute adjusted risk (%) at 3 years^a^, women vs men	Risk difference (females − males) (95% CI); *P*-value	Unadjusted HR (95% CI), women vs men	Adjusted HR^b^ (95% CI), women vs men
KRT initiation	1930 (20.6)	17.9% (17.1–18.6)	−4.5%-units (−5.5 to −3.5)	18.0% (17.1–18.6)	−4.8%-units (−5.8 to −3.9)	**0.76 (0.72–0.80)**	**0.75 (0.70–0.80)**
	3984 (25.1)	22.4% (21.6–23.2)	***P* < .001**	22.8% (22.0–23.1)	***P* < .001**		
Dialysis initiation	1799 (19.2)	16.7% (16.0–17.3)	−4.2%-units (−5.1 to −3.3)	16.6% (15.8–17.3)	−4.4%-units (−5.4 to −3.5)	**0.75 (0.71–0.80)**	**0.75 (0.70–0.80)**
	3739 (23.5)	20.9% (20.2–21.5)	***P* < .001**	21.0% (20.3–21.6)	***P* = .001**		
Dialysis initiation in planned access	788 (8.4)	7.7% (7.2–8.2)	−2.0%-units (−2.6 to −1.3)	8.7% (8.1–9.3)	−2.6%-units (−3.4 to −1.7)	**0.84 (0.81–0.88)**	**0.82 (0.78–0.85)**
	1598 (10.1)		***P* < .001**	11.3% (10.6–11.8)	***P* = .001**		
Preemptive kidney transplantation	131 (1.4)	1.1% (0.9–1.3)	−0.2%-units (−0.4 to 0.1)	1.2% (1.0–1.5)	−0.2%-units (−0.5 to 0.1)	0.81 (0.66–1.01)	0.84 (0.67–1.05)
	245 (1.5)	1.2% (1.0–1.2)	*P* = .21	1.4% (1.2–1.6)	*P* = .06		
LD preemptive	75 (0.8)	0.7% (0.5–0.8)	−0.1%-units (−0.3 to 0.0)	0.8% (0.6–1.0)	−0.1%-units (−0.4 to 0.1)	0.78 (0.59–1.03)	0.82 (0.61–1.10)
	148 (0.9)	0.8 (0.7–1.0)	*P* = .15	0.9% (0.8–1.1)	*P* = .18		
Death before KRT initiation	2141 (22.9)	20.1% (19.2–20.8)	−2.7%-units (−3.6 to −1.9)	20.9% (19.9–21.6)	−2.8%-units (−3.9 to −2.0)	**0.83 (0.79–0.88)**	**0.81 (0.77–0.85)**
	4017 (25.2)	22.8% (21.9–23.4)	***P* < .001**	23.7% (23.0–24.5)	***P* < .001**		
CKM decision	429 (4.7)	4.6% (4.1–5.0)	0.7%-units (0.1–1.2)	3.7% (3.3–4.1)	0.3%-units (−0.1 to 0.7)	1.13 (1.00–1.28)	1.05 (0.92–1.20)
	590 (3.8)	3.9% (3.6–4.2)	***P* = .02**	3.4% (3.1–3.7)	*P* = .16		

The table presents absolute risk of the KRT-related events as well as HR. The absolute risk was predicted at 3 years with flexible parametric regression and 95% CI calculated with bootstrapping, 200 repetitions. This risk was presented unadjusted as well as adjusted. HR were calculated using cause-specific Cox proportional hazards regression. Patients were followed from inclusion until KRT initiation, CKM decision, death or 31 December 2020, which ever occurred first. Missing data were handled using multiple imputation by chained equations in Stata, generating 20 imputed datasets with linear regression models.

Adjusted for sex (legal gender), age, eGFR-splines (5 knots), hypertension, congestive heart failure, diabetes mellitus, ischaemic heart disease, stroke, vascular disease. CKM decision also adjusted for a diagnosis of cancer the last 5 years and dementia.

Adjusted for sex (legal gender), age-splines, eGFR-splines, pulse pressure, ACR (logarithmic), haemoglobin (logarithmic), phosphate (logarithmic), hypertension, congestive heart failure, diabetes mellitus, ischaemic heart disease, stroke, vascular disease. CKM decision also adjusted for a diagnosis of cancer the last 5 years and dementia.

b Bold values have significant confidence intervals

### Hazard ratios

The results from the cause specific cox regression analysis were consistent with the already presented absolute risks, showing that women were less likely to initiate KRT (HR 0.75, 95% CI 0.70–0.80) (Table 3). Female gender was associated with lower rate of dialysis initiation (0.75, 0.70–0.80) and of initiating dialysis with a planned access (0.82, 0.78–0.85). There were no significant gender differences regarding preemptive kidney transplantation, including LD transplantation. Female gender was associated with lower rate of death before KRT (0.81, 0.77–0.85). The absolute unadjusted risk difference in CKM decisions (more women choosing CKM) was translated into borderline significant HR unadjusted (1.13, 1.00–1.28) but no significant difference after adjustments (1.05, 0.92–1.20). Figure 1B summarizes these results.

### Sensitivity analyses

The sensitivity analyses with complete cases (*n* = 13 196 ) and two different time periods, 2010–15 (*n* = 13 568) and 2016–20 (*n* = 11 910), show results aligning with the main analysis, with some exceptions; the gender difference in RAASi and BP control at index was mitigated in the later time period (2015–20) and the gender difference in anticoagulants was no longer seen in the complete cases analysis (Supplementary data, Table S4). The gender difference in initiation of dialysis in planned access was no longer seen in the later time period (2016–20) and in the complete cases analysis women had lower chances of LD transplantation (Supplementary data, Table S5). Results are summarized in Supplementary data, Figs S1 and S2. The post-hoc analysis investigating time to KRT-related events from documented KRT decision was consistent with the main findings except for the outcome death before KRT, where the there was no longer a gender difference (Supplementary data, Table S6). Expanding the CKD diagnosis by more ICD-10 codes increased the percentage of patients who had a documented CKD diagnosis from 93% to 95%, however it did not mitigate the gender difference—women still had lower chances of having a CKD diagnosis (OR 0.85, 95% CI 0.74–0.97) (Supplementary data, Table S7).

## DISCUSSION

The importance of gender equity in CKD management is being increasingly recognized. Using data from a nationwide, large cohort of CKD patients, we hypothesized that women under nephrology care are less likely to receive recommended CKD management and guideline-recommended initiation of KRT including preemptive kidney transplantation. Overall, our study supports this hypothesis by showing that women were underdiagnosed and undertreated with guideline-recommended therapies at the time when eGFR dropped below 30 mL/min/1.73 m^2^. During the course of nephrology care, the outcomes displayed more heterogenous results, where women had lower risk of starting KRT and of dying before KRT, equal chances of preemptive kidney transplantation and CKM decision, but lower chance of initiating dialysis with a planned access. These gender differences may be partly attributable to biological factors, including the burden of comorbidities and CKD progression rates; however, gender-related factors may also be present.

Despite higher prevalence of CKD in women, the present study shows that only one-third of patients in Swedish nephrology care with eGFR <30 mL/min/1.73 m^2^ are women. Male dominance in nephrology clinics is also seen in other countries and a plausible explanation is the more albuminuric progressive disease in men [21]. However, gender-related factors may also contribute. A Swedish study found that women meeting eGFR and albuminuria criteria were less often accepted for nephrology referral [6]. The finding that women in the present study are entered into SRR-CKD later than men probably reflects the same pattern. Thus, the lower probability of having eGFR >30 mL/min/1.73 m^2^ at first registration in SRR-CKD is likely because women are seen by nephrologists for the first time at a lower eGFR, rather than a systematic bias in the registry. An explanation for the later referrals could be that CKD in women is not acknowledged by primary care. The relatively low creatinine in combination with a low eGFR, naturally seen in women due to lower body mass, might deceive the physician into overestimating the kidney function, leading to missed CKD diagnosis and delayed or not accepted nephrology referral. This is supported by our finding that women with eGFR <30 mL/min/1.73 m^2^ are less likely to have a documented CKD diagnosis, regardless of whether we used an N18 diagnosis (as an indicator of CKD recognition) or a specified kidney disease diagnosis as in the sensitivity analysis. Other potential explanations to late referral include women seeking medical attention less or later due an inability to prioritize their health [22].

In the present study, women were less often treated with RAASi, a pattern previously observed in both Europe and North America [8, 23]. An explanation for this might be late CKD recognition and late referral of women leading to reluctancy to initiate RAASi once referred, due to low GFR or women not tolerating RAASi treatment. Other explanations could be lack of RAASi indication due to low ACR, or less hypertension, diabetes and heart failure in females. However, this does not fully explain the gender difference since comorbidities and albuminuria were adjusted for in the present study. It is crucial to consider whether gender bias is involved, with a perception that RAASi is less suitable or necessary in women, especially since the study suggests that women less often achieve BP target. The present study suggests that women additionally have less guideline-recommended treatment from cardiovascular disease prevention such as undertreatment with lipid-lowering agents, which according to KDIGO 2024 guidelines should be prescribed in all patients with eGFR <60 mL/min/1.73 m^2^. Furthermore, our study shows that women with AF had lower chances of having anticoagulant treatment, even though it is proven safe and effective in CKD G3–G4 irrespective of sex [24–27].

There were borderline-significant differences in the adjusted odds of having a kidney biopsy among nephrology-referred patients with advanced CKD. Considering the skewness in access to nephrology care, with approximately one-third of patients with eGFR <30 mL/min/1.73 m^2^ being women despite women having more prevalent CKD in Sweden, this results in overall better access to biopsies in men and subsequent possible better disease awareness and targeted treatment.

The present study demonstrates that female gender is associated with a 25% lower risk of initiating KRT, translating to 67% KRT initiations occurring in men. This male predominance aligns with previous literature, where >60% of incident KRT patients in Europe and the USA are men [28, 29]. Men generally experience a faster CKD progression, which likely contributes the most to the higher KRT rates; however, gender-related factors may also contribute. The post-hoc analysis investigating time-to-event from KRT decision suggest that there might be other factors involved, since even though a KRT decision is documented, women still have lower chances of initiating KRT. In addition, prior studies recognized that women initiate dialysis at lower eGFR than men despite reporting more symptoms [30–32]. Underdiagnosis and lower referral rates to nephrology among women may contribute; additionally, more women choosing CKM has been proposed as part of the explanation to lower KRT rates [3, 33]. In our study, women displayed higher unadjusted absolute risk of having a CKM decision but no difference in adjusted risk, potentially reflecting that other factors influence treatment decision, such as age—older women often consider themselves more frail than age-matched men and are often more positive to palliative approaches [34, 35]. As the survival benefit of dialysis in older patients remains uncertain, potential gender differences cannot be evaluated regarding appropriateness, but warrant awareness when supporting patients in treatment pathways [36, 37].

The lower risk of KRT initiation in women observed in the present study is mainly driven by lower dialysis initiation rates. There is no apparent gender difference regarding risk of preemptive kidney transplantation, including LD transplants. This should be interpreted with caution due to the low number of transplantations, but the results align with contemporary data from the European Renal Registry [28] and are encouraging since many studies have shown disproportionately fewer waitlisted and transplanted women [38]. A US study found no gender differences in the likelihood of being referred to preemptive kidney transplantation; however, the transplant-referred women had a more favourable clinical profile, leading the authors to propose that women need to meet a higher threshold for referral [39]. This theory was supported by others reporting that older women have lower chances of being transplanted than same-age men [40, 41]. Investigating gender disparities in transplantation is complex since more men progress to KRT—it follows that more men need kidney transplants. In addition, biological factors such as sensitization can result in longer waiting times for women [42]. Data from the present study did not include patients transplanted after KRT initiation and do not give the full picture on gender differences in kidney transplant access.

The present study found that women are less likely to initiate dialysis with a planned access. Similar findings have been reported in Europe and the USA, where female sex was identified as a risk factor for initiating haemodialysis with a catheter [43–45]. Possible explanations include anatomical differences and greater female patient reluctancy towards arteriovenous fistula surgery. In addition, gender disparities related to race and late referral have been associated with reduced likelihood of a preplanned access [46].

Given that men with CKD are more likely to die and initiate KRT than women, one might argue that the gender disparities are acceptable—after all, women appear to fare better than men despite receiving less guideline-recommended management. Indeed, it is yet to be proven whether improved recognition and management of CKD translate into better outcomes. An understudy to the Chronic Renal Insufficiency Cohort (CRIC) Study found that lack of nephrologist contact was associated with less RAASi prescription but not with CKD progression, cardiovascular disease or death [47]. However, determining whether women should truly be managed differently from men requires greater inclusion of women with CKD in randomized controlled trials, which first necessitates better recognition of female CKD. A key question is also whether women with CKD should be compared with men, or rather with other women. Females with CKD experience higher mortality and morbidity compared with women without or with lower grade of CKD [48]. Our data from the post-hoc analysis support this, showing that the survival benefit seen in women was diminished when the analysis was limited from KRT decision to death. Framing it this way might help to reinforce the argument that women with CKD should, of course, have equal access to recommended therapy to reach their full health potential.

A strength of this study is the high validity and coverage of the SRR-CKD together with Swedish the tax-funded healthcare, which is highly accessible for all citizens. The limitations with register cohorts involve several biases and confounders, including misregistration and internal validity issues. The results cannot be generalized to all Swedish patients with advanced CKD since patients cared for in primary care are not included in the SRR-CKD.

## CONCLUSIONS

Swedish women with incident eGFR <30 mL/min/1.73 m^2^ attending specialized nephrology care remain underdiagnosed, undertreated and less likely to reach BP targets. Although these gender differences did not translate to increased KRT and mortality rates, they highlight the need for nephrologists to maintain awareness and strive for health equity.

## Supplementary Material

sfag302_Supplemental_File

## Data Availability

The data underlying this article will be shared on reasonable request to the corresponding author.
