# Supplementary material for: Gender disparities in Swedish specialized nephrology care
Source: Clin Kidney J. 2026 Sep 1;19(10):sfag302. doi: 10.1093/ckj/sfag302 (PMC13627835; doi:10.1093/ckj/sfag302)
Supplement: Supplementary file 1 [file sfag302_supplemental_file.docx]

**Supplement:**

**Table S1. Diagnoses of primary kidney diseases not requiring a kidney biopsy and therefor excluded from the kidney biopsy analysis:**

| PRD-EDTA codes | 1057 ,1061 ,1298 ,1279 ,1074 ,1035 ,1602 ,1618 ,1625 ,1639 ,1641 ,3627 ,1660 ,1673 ,1687 ,1694 ,1706 ,1710 ,1723 ,1752 ,1768 ,1775 ,1781 ,1799 ,1809 ,1813 ,1821 ,3689 ,3670 ,1907 ,2226 ,2453 ,2469 ,2476 ,2521 ,2668 ,2675 ,2718 ,2725 ,2739 ,2741 ,2756 ,2794 ,2804 ,2815 ,2827 ,2843 ,2858 ,2862 ,2870 ,2889 ,2891 ,2901 ,2929 ,2938 ,2955 ,2964 ,2972 ,2986 ,2993 ,3000 ,3016 ,3028 ,3037 ,3044 ,3059 ,3063 ,3071 ,3085 ,3092 ,3194 ,3207 ,3211 ,3731 ,3276 ,3282 ,3253 ,3295 ,3305 ,3248 ,3314 ,3658 ,3322 ,3333 ,3346 ,3351 ,3367 ,3461 ,3517 ,3529 ,3538 ,3540 ,1747 ,3783 ,3796 ,3806 |
| --- | --- |

**Table S2. List of ICD-10 codes used for extracting covariates from NPR**

| **Baseline comorbidities or covariates** | **ICD-10** |
| --- | --- |
| Atrial fibrillation | I48 |
| Cancer | C00-C26, C30-C41, C43, C45-C58, C60-C97 |
| Chronic kidney disease | N18 |
| Chronic obstructive pulmonary disease or asthma | J43, J44, J45 |
| Congestive heart failure | I110, I130, I132, I50 |
| Dementia | F00-F03 |
| Diabetes | E10-14 |
| Hypertension | I10-I13, I15 |
| Ischemic heart disease | I20-I23, I241, I248, I249, I251, I252, I255, I256, I258, I259 |
| Major bleeding | I85.0, I98.3, K226, K250, K252, K254, K256, K260, K262, K264, K266, K270, K272, K274, K276, K280, K282, K284, K286, K625, K661, K920-K922, I60-I62, S064-S066, H313, H356, H431, H450, I312, J942, M250, N501A N939, N950, R04, R319, R319A, R589, T810, D629 |
| Myocardial infarction | I21, I22, I252 |
| Psychiatric disease | F20-F29, F30-F34, F41 |
| Stroke | I63, I64, I69 |
| Systemic embolism | I74 |
| Vascular disease | I21, I22, I252, I70-I73, I65 |
| Venous thromboembolism | I26, I82 |

**Table S3. Table on missingness of clinical baseline characteristics extracted from SRR-CKD**

| **Variable** | **Missing n (%)** |
| --- | --- |
| ACR | 11064 (43.4%) |
| Phosphate | 2186 ( 8.6%) |
| Haemoglobin | 876 ( 3.4%) |
| Pulse pressure | 1423 ( 5.6%) |

**Table S4. Sensitivity analyses, Gender differences in CKD management at index**

|  | **Adjusted Odds ratios (95%CI)**  N= 25478  ***MAIN ANALYSIS***  (multiple imputation) | **Complete cases,** **adjusted OR**  N= 13196 | **2010-2015**  **Adjusted OR**  (multiple imputation)  N=13568 | **2016-2020**  **Adjusted OR**  (multiple imputation)  N=11910 |
| --- | --- | --- | --- | --- |
| Documented CKD diagnosis (N18) | **0.81 (0.73-0.90)** | **0.83 (0.72-0.96)** | **0.86 (0.75-0.97)** | **0.73 (0.62-0.86)** |
| RAASi | **0.94 (0.89-0.99)** | **0.91 (0.84-0.98)** | **0.92 (0.85-0.99)** | 0.97 (0.89-1.05) |
| Lipid-lowering agent | **0.93 (0.88-0.99)** | **0.91 (0.84-0.99)** | 0.96 (0.88-1.04) | **0.91 (0.84-0.99)** |
| Anticoagulants if AF | **0.86 (0.76-0.98)** | 0.97 (0.80-1.17) | **0.80 (0.67-0.97)** | 0.94 (0.77-1.14) |
| Diagnosis by kidney biopsy | 0.93 (0.86-1.00) | 0.94 (0.85-1.05) | 0.88 (0.75-1.03) | 0.96 (0.86-1.07) |
| BP <140/90 | **0.91 (0.86-0.97)** | **0.82 (0.76-0.90)** | **0.89 (0.82-0.98)** | 0.93 (0.85-1.02) |
| Early entry in SRR-CKD, first eGFR ≥30 ml/min/1.73m^2^ | **0.78 (0.72-0.84)** | **0.75 (0.69-0.82)** | **0.78 (0.70-0.87)** | **0.78 (0.71-0.86)** |

*The table presents results from the main analysis (using multiple imputation) along side with three sensitivity analyses regarding gender differences (women versus men) incident patients at the first outpatient visit with eGFR<30ml/min/1.73m^2^ registered in SRR-CKD in complete cases (no imputed data) as well as analyses from two index time periods (2010-2015 and 2016-2020), using imputed data. Results presented with odds ratios and 95% confidence interval. Analyses are* *adjusted for: sex, age, eGFR, pulse pressure, ACR (logarithmic), hypertension, congestive heart failure, diabetes mellitus, ischemic heart disease, stroke, vascular disease. Anticoagulant treatment is also adjusted for haemoglobin (logarithmic) and previous major bleeding. Kidney biopsy is also adjusted for anticoagulant treatment, previous major bleeding and haemoglobin (logarithmic).*

**Table S5. Sensitivity analyses, Gender differences in initiation of KRT**

| Outcome | Female vs male sex  Adjusted Hazard Ratio (95% confidence interval)  Multiple imputation (main analysis) | Complete cases  Adjusted Hazard Ratio (95% confidence interval) | 2010-2015  Adjusted Hazard Ratio (95% confidence interval)  Multiple imputation | 2016-2020  Adjusted Hazard Ratio (95% confidence interval)  Multiple imputation |
| --- | --- | --- | --- | --- |
| KRT initiation | **0.75 (0.70-0.80)** | **0.73 (0.67-0.80)** | **0.75 (0.69-0.81)** | **0.85 (0.77-0.94)** |
| Dialysis initiation | **0.75 (0.70-0.80)** | **0.73 (0.67-0.80)** | **0.74 (0.69-0.81)** | **0.75 (0.67-0.84)** |
| Dialysis initiation in planned access | **0.82 (0.78-0.85)** | **0.84 (0.79-0.89)** | **0.74 (0.66-0.82)** | 0.84 (0.70-1.06) |
| Preemptive kidney transplantation | 0.84 (0.67-1.05) | 0.75 (0.55-1.03) | 0.86 (0.66-1.12) | 0.83 (0.55-1.27) |
| Living Donor Preemptive | 0.82 (0.61-1.10) | **0.62 (0.40-0.96)** | 0.89 (0.63-1.26) | 0.68 (0.38-1.22) |
| Death before KRT initiation | **0.81 (0.77-0.85)** | **0.85 (0.79-0.93)** | **0.78 (0.74-0.81)** | **0.85 (0.78-0.94)** |
| CKM decision | 1.05 (0.92-1.20) | 1.07 (0.89-1.28) | 1.03 (0.87-1.22) | 1.07 (0.87-1.32) |

*The table presents three sensitivity analyses regarding gender differences in KRT-related events; complete cases as well as analyses from two index time periods (2010-2015 and 2016-2020). The analyses show gender differences in KRT-related events (women versus men) with hazard ratios and 95% confidence interval from a fully adjusted cause-specific Cox proportional hazards regression analysis. Adjusted models include sex (legal gender), age and eGFR (cubic splines, 5 knots), pulse pressure and logarithmic (log) ACR (modelled linearly), hypertension, congestive heart failure, diabetes mellitus, ischemic heart disease, stroke and vascular disease. The outcome anticoagulant treatment was also adjusted for log-haemoglobin (linearly) and history of a previous major bleeding. The outcome kidney biopsy was additionally adjusted for anticoagulant treatment, previous major bleeding and log-haemoglobin.* *Patients were followed from inclusion until KRT initiation, CKM decision, death or December 31, 2020, which ever occurred first.*

**Table S6.** Gender differences in time to KRT-related outcomes from treatment decision

| Outcome | Women  n, (% of eligible)  Men  n, (% of eligible) | Women vs men  Unadjusted hazard Ratio (95% confidence interval) | Women vs men  Adjusted Hazard Ratio (95% confidence interval) |
| --- | --- | --- | --- |
| KRT initiation | 1004 (64.2)  2119 (68.9) | **0.83 (0.77-0.90)** | **0.84 (0.77-0.91)** |
| Dialysis initiation | 937 (60.0)  1997 (64.9) | **0.83 (0.77-0.90)** | **0.84 (0.77-0.91)** |
| Dialysis initiation in planned access | 730 (46.7)  1472 (47.9) | **0.88 (0.80-0.96)** | **0.87 (0.80-0.96)** |
| Preemptive kidney transplantation | 68 (4.3)  124 (4.0) | 0.92 (0.68-1.24) | 0.96 (0.70-1.32) |
| Living Donor Preemptive | 37 (2.3)  82 (2.7) | 0.77 (0.52-1.14) | 0.84 (0.55-1.28) |
| Death before KRT initiation | 167 (10.7)  318 (10.3) | **0.88 (0.73-1.06)** | 0.97 (0.79-1.18) |

*Table S6 shows gender differences in time to KRT-related outcomes from treatment decision to start of any KRT in 4639 patients (1563 females and 3076 males). Median follow up time for women was 0.87 years (IQR 0.34-1.88) and for males 0.72 years (0.28-1.59). Note that this analysis only includes patients with a documented decision, not all patients with a decision to start KRT will start KRT and many patients who start KRT do not have a previously documented decision.*

**Table S7** Sensitivity analysis defining CKD diagnosis as any documented ICD-10 diagnosis of N00-N19 and Q61 at or prior index

|  | Women  n, (% of eligible)  Men  n, (% of eligible) | **Unadjusted Odds Ratio**  (95% confidence interval) | **Adjusted Odds ratios**  (95% confidence interval) |
| --- | --- | --- | --- |
| Documented CKD diagnosis (N18)  Data from main analysis for comparison | 8661 (92%)  14985 (93%) | **0.78 (0.71-0.86)** | **0.81 (0.73-0.90)** |
| CKD diagnosis (N00-N19 and Q61) at index | 9016 (95.5%)  15466 (96.4%) | **0.77 (0.69-0.89)** | **0.85 (0.74-0.97)** |

*Table S7 is a sensitivity analysis investigating the robustness regarding gender differences (women versus men) in a documented CKD diagnosis at index, adding all ICD-10 codes N00-N19 and Q61 to the N18 diagnosis. Results presented with odds ratios and 95% confidence interval. Analyses are* *adjusted for: sex (legal gender), age, eGFR, pulse pressure, ACR (logarithmic), hypertension, congestive heart failure, diabetes mellitus, ischemic heart disease, stroke, vascular disease.*

**Figure S1**

*Figure S1 presents three sensitivity analyses regarding gender differences (women versus men) incident patients at the first outpatient visit with eGFR<30ml/min/1.73m^2^ registered in SRR-CKD in complete cases as well as analyses from two index time periods (2010-2015 and 2016-2020). Results presented with odds ratios and 95% confidence interval. Adjusted models include sex (legal gender), age and eGFR (cubic splines, 5 knots), pulse pressure and logarithmic (log) ACR (modelled linearly), hypertension, congestive heart failure, diabetes mellitus, ischemic heart disease, stroke and vascular disease. The outcome anticoagulant treatment was also adjusted for log-haemoglobin (linearly) and history of a previous major bleeding. The outcome kidney biopsy was additionally adjusted for anticoagulant treatment, previous major bleeding and log-haemoglobin.*

**Figure S2**

***Figure S2*** *presents three sensitivity analyses regarding gender differences in KRT-related events; complete cases as well as analyses from two index time periods (2010-2015 and 2016-2020). The analyses show gender differences in KRT-related events (women versus men) with hazard ratios and 95% confidence interval from a fully adjusted cause-specific Cox proportional hazards regression analysis. Adjusted for sex, age-splines, eGFR-splines, pulse pressure, ACR (logarithmic), haemoglobin (logarithmic), phosphate (logarithmic), hypertension, congestive heart failure, diabetes mellitus, ischemic heart disease, stroke, vascular disease. Conservative care also adjusted for a diagnosis of cancer the last 5 years and dementia. Patients were followed from inclusion until KRT initiation, CKM decision, death or December 31, 2020, which ever occurred first.*

**Figure S3 a-g, larger copies of the figures 2a-g from the main manuscript:**

Figure S3a Time to KRT initiation

p<0.001

Figure S3b Time do dialysis initiation

 p<0.001

Figure S3c Time to KRT initiation with planned access

p<0.001

Figure S3d Time to preemptive transplantation

p=0.17

Figure S3e Time to living donor transplantation

P=0.16

Figure S3f Time to death before KRT initiation

 p<0.001

Figure S3g Time to CKM decision

P=0.004
